# Transcatheter closure of an ascending aortic pseudoaneurysm using an atrial septal defect occluder: a case report

**DOI:** 10.1186/s43044-025-00663-x

**Published:** 2025-06-16

**Authors:** Georgios E. Papadopoulos, Ilias Ninios, Sotirios Evangelou, Andreas Ioannides, Vlasis Ninios

**Affiliations:** Cardiology Department, Interbalkan Medical Center, Thessaloniki, Greece

**Keywords:** Ascending aortic pseudoaneurysm, Transcatheter closure, Atrial septal defect occlude, Structural heart intervention, Case report

## Abstract

**Background:**

Ascending aortic pseudoaneurysm is a rare but potentially fatal complication following cardiac surgery. Its management is particularly challenging in elderly or frail patients due to the high risk associated with repeat sternotomy. This case is notable for the successful use of a transcatheter approach to treat a pseudoaneurysm arising just above a sutureless aortic valve bioprosthesis—an unusual anatomical location that poses unique technical challenges.

**Case presentation:**

An 83-year-old man with a history of hypertension underwent surgical aortic valve replacement with a large-size sutureless bioprosthetic valve. Five months later, he presented with chest heaviness. Imaging with computed tomography angiography and aortography revealed an ascending aortic pseudoaneurysm located 1.5 cm above the upper edge of the prosthetic valve frame. Due to his advanced age and stable clinical condition, a percutaneous approach was selected. Access was obtained via the right femoral artery, and angiography was performed using a pigtail catheter. An initial attempt to close the defect with a 22 mm atrial septal defect occluder failed due to inadequate anchoring. A 32 mm device was then successfully deployed, sealing the pseudoaneurysm without interfering with valve function. The patient remained hemodynamically stable throughout the procedure and had an uneventful recovery. Follow-up imaging at six months confirmed stable device positioning, complete exclusion of the pseudoaneurysm from systemic circulation, and no thrombus formation. The patient remained asymptomatic.

**Conclusions:**

This case demonstrates that transcatheter closure of ascending aortic pseudoaneurysms is a viable and safe alternative to surgical repair in selected high-risk patients. The successful use of an atrial septal defect occluder above a sutureless aortic valve prosthesis highlights the adaptability of percutaneous closure devices and the importance of individualized procedural planning. This approach may expand treatment options for patients otherwise considered inoperable.

**Supplementary Information:**

The online version contains supplementary material available at 10.1186/s43044-025-00663-x.

## Background

Aortic pseudoaneurysm (AP) represents a life-threatening vascular abnormality characterized by a focal outpouching of the aortic wall, contained solely by periaortic connective tissue. When occurring in the ascending aorta (AAP), these pseudoaneurysms are most commonly observed as a rare but severe complication following cardiac or aortic surgery. Other etiologies, including trauma, infection, and inflammatory conditions, can also contribute to their formation. Due to their potential for catastrophic complications—such as rupture, thromboembolism, and fistula formation—AAPs require prompt diagnosis and definitive management [1].

The incidence of AAPs remains difficult to establish, with reported rates ranging from 0.5 to 13% in post-cardiac surgery patients. While many remain asymptomatic, progressive enlargement can result in compressive symptoms, including chest pain, dyspnea, and syncope. Without intervention, mortality rates range between 29 and 46%, with exsanguination from rupture being the most frequent cause of death [2]. Conservative management has historically yielded poor outcomes, with mortality reaching up to 61% [3]. Given the significant risks associated with untreated pseudoaneurysms, definitive repair is always indicated.

Surgical intervention remains the gold standard for AAP management, demonstrating long-term efficacy in reducing mortality. However, its complexity—often requiring redo sternotomy and cardiopulmonary bypass—contributes to an in-hospital mortality rate of 6.9%–12.6% [4]. Additionally, the high morbidity, prolonged recovery, and increased healthcare costs associated with surgical repair underscore the need for alternative treatment strategies. Endovascular approaches, particularly stent grafting, have emerged as less invasive alternatives but are not without limitations. Reported all-cause mortality following endovascular repair is approximately 15.2%, with aorta-related mortality at 5%, type I endoleak rates of 18.6%, and a reintervention rate of 9.3% [5]. Moreover, conventional stent graft placement is constrained by the need for branch-free landing zones or the use of complex fenestrated devices, further complicating their applicability in AAP cases.

In response to these challenges, novel percutaneous techniques, including vascular plug and occluder-based approaches, have gained interest as potential solutions for selected patients. While evidence remains limited, early reports suggest that these devices may offer a safe, effective, and durable alternative for pseudoaneurysm closure in cases where open surgery is high risk or contraindicated [1]. This report describes a case where a transcatheter approach using an ASD occluder was utilized to successfully treat an ascending aortic pseudoaneurysm.

## Case presentation

An 83-year-old male with a history of hypertension underwent surgical aortic valve replacement (SAVR) with a Perceval S large-size prosthesis. His postoperative course was uneventful. However, five months postoperatively, he presented with complaints of chest heaviness. A routine chest X-ray revealed mediastinal widening, prompting further investigation.

The operative report documented an ascending aortic diameter of 38 mm at the sinotubular junction, tapering to 34 mm at the mid-ascending segment, with an aortic wall thickness of approximately 2.0 ± 0.2 mm, with focal calcific plaques predominantly along the greater curvature but no areas of friable or thinned tissue. The native aorta exhibited mild medial degeneration consistent with age-related changes but preserved tensile integrity. A standard transverse aortotomy was closed in two layers using 4–0 polypropylene mattress sutures buttressed with polyethylene felt strips. No intraoperative evidence of suture line bleeding or dehiscence was noted.

Computed tomography angiography (CTA) confirmed the presence of an AAP located 1.5 cm above the upper limit of the prosthetic valve frame. The etiology of the AAP in this case appears most consistent with late dehiscence at the prosthesis–aortic interface, rather than direct iatrogenic injury from aortic cannulation or the aortotomy. The pseudoaneurysm originated 1.5 cm above the upper edge of the prosthetic valve frame, distant from the reported cannulation site, which was located more proximally along the anterior curvature of the ascending aorta. Intraoperative records described an uncomplicated cannulation and decannulation without evidence of trauma or bleeding. Furthermore, the localization of the pseudoaneurysm near the prior aortotomy closure line suggests that mechanical stress at the suture line—possibly exacerbated by postoperative hypertension or focal tissue fragility—may have contributed to progressive weakening and disruption.

**Fig. 1:**
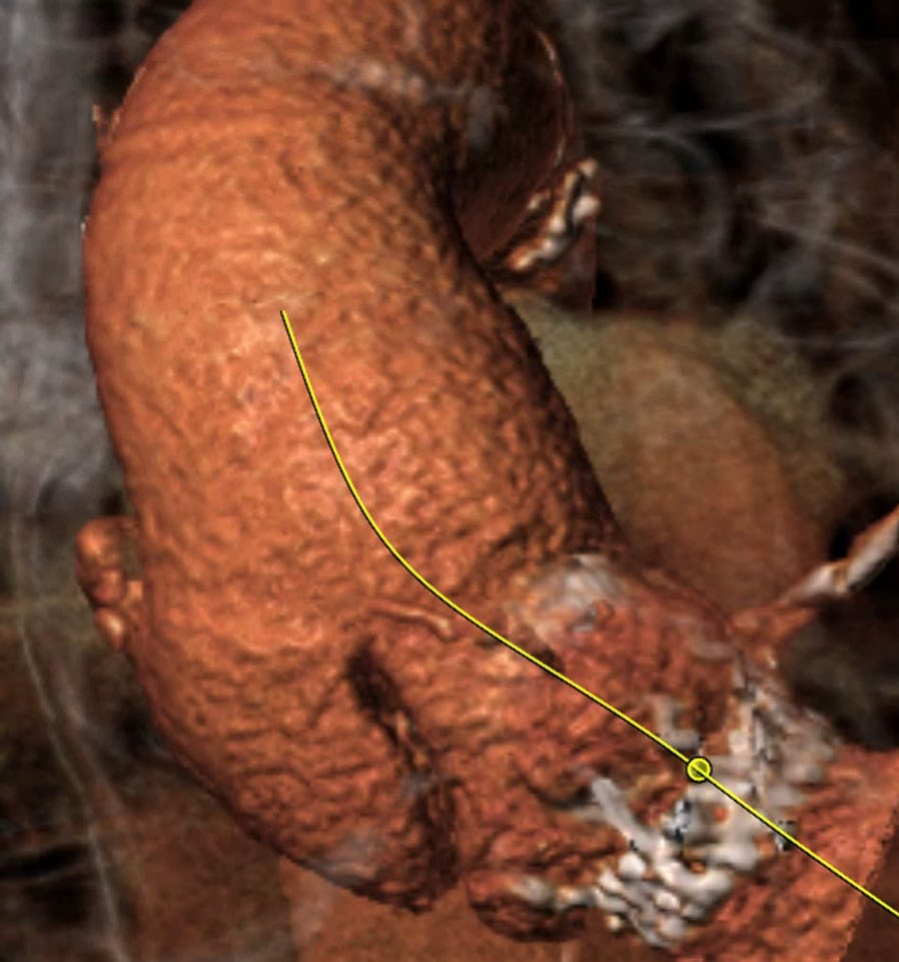
Three-dimensional CT reconstruction image

A three-dimensional reconstruction provided a detailed anatomical delineation of the defect (Fig. 1). The diagnosis was further confirmed by aortography, which offered dynamic assessment of the pseudoaneurysm and its flow characteristics. Given the patient’s advanced age and relative clinical stability, a transcatheter approach was considered as an alternative to high-risk open surgery.

**Fig. 2:**
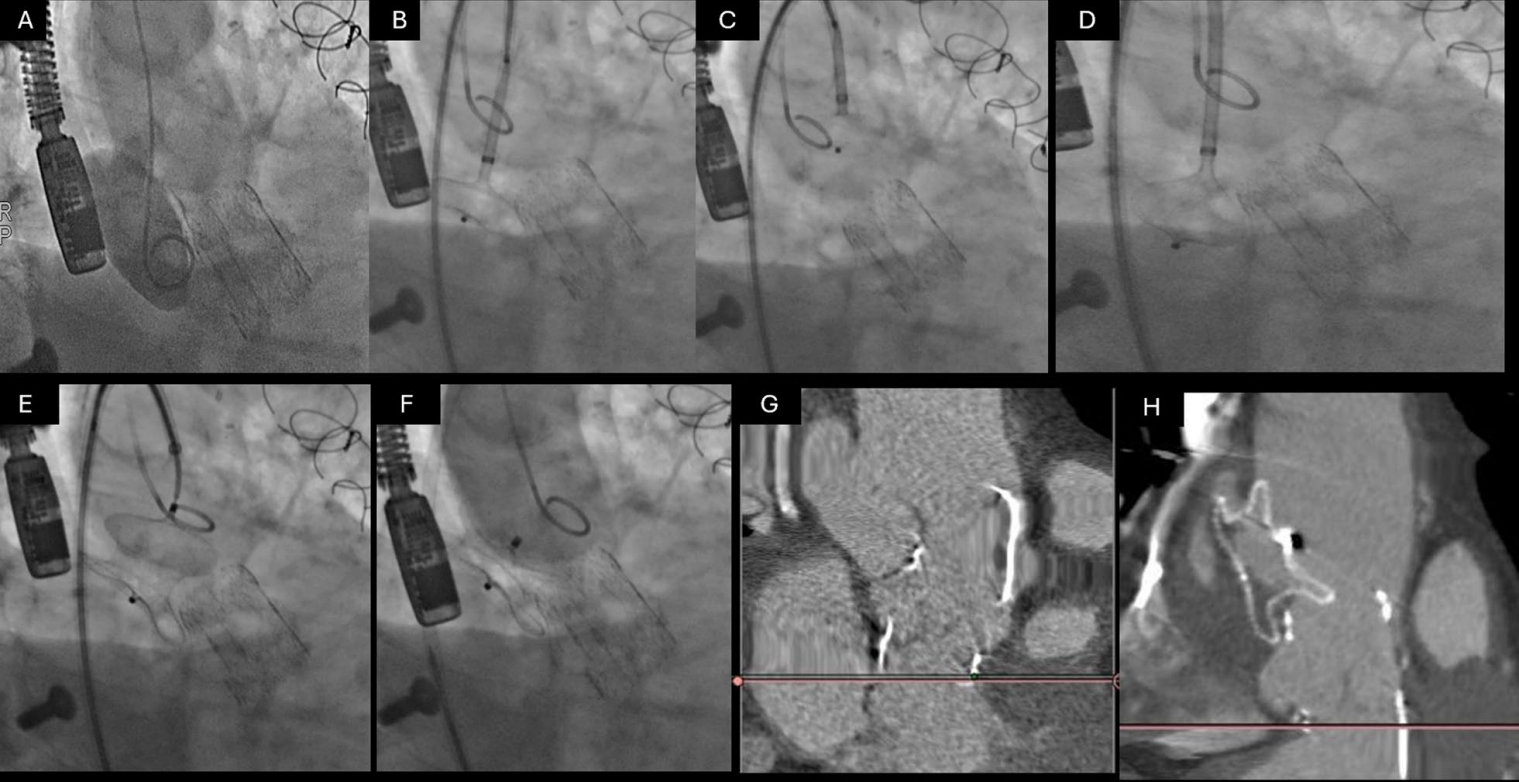
Percutaneous Closure of Ascending Aortic Pseudoaneurysm using an Atrial Septal Defect (ASD) Occluder. **A** Initial angiographic assessment showing the pseudoaneurysm located above the prosthetic valve frame. **B** Introduction of a nine-French atrial septal defect (ASD) delivery system after successful guidewire placement. **C** Initial attempt with a 22 mm ASD occluder, which failed to anchor properly as the distal disk passed through the aneurysm neck without resistance. **D** Successful device placement using a 32 mm ASD occluder, providing adequate sealing of the pseudoaneurysm. **E** Angiographic confirmation of the correct position of the occluder with no obstruction to the prosthetic valve. **F** Final angiogram demonstrating complete exclusion of the pseudoaneurysm from systemic circulation, with no evidence of device migration

The procedure was performed via right femoral artery access using a six-French sheath. A pigtail catheter was advanced into the pseudoaneurysm to facilitate angiographic assessment and precise device placement (Fig. 2A, Supplementary Video). The system was then exchanged for an Amplatz super-stiff wire, and a nine-French atrial septal defect delivery system was introduced (Fig. 2B). An initial attempt with a 22 mm atrial septal defect occluder (ASD) failed to achieve adequate anchoring as the distal disk opened and was pulled through the aneurysm neck without resistance (Fig. 2C, Supplementary Video). In response, the device was upsized to a 32 mm atrial septal defect occluder, which provided a satisfactory fit with adequate sealing of the aneurysm (Fig. 2D,E,F, Supplementary Video). Importantly, there was no evidence of obstruction to the prosthetic valve, and angiographic assessment confirmed successful exclusion of the pseudoaneurysm from systemic circulation.

The patient remained hemodynamically stable throughout the procedure and experienced an uneventful recovery. Immediate post-procedural imaging confirmed appropriate device placement, complete exclusion of the pseudoaneurysm, and the absence of thrombus formation. A follow-up computed tomography scan six months later showed good sealing of the defect, nearly complete thrombosis of the pseudoaneurysm, and no evidence of device migration (Fig. 2G, H). The patient remained asymptomatic, with no recurrence of chest discomfort or cardiovascular.

## Conclusions

In retrospect, more aggressive debridement of aortic wall calcification and the use of fully interrupted, pledgeted sutures may have reduced focal stress at the aortotomy site. Additionally, biologic buttressing material might have allowed better tissue integration and decreased long-term tension at the suture–aorta interface. Transcatheter closure using an ASD occluder represents a feasible, safe, and minimally invasive alternative for treating ascending aortic pseudoaneurysms in high-risk surgical patients. Precise sizing of the aneurysm neck remains a challenge, and multimodal imaging should be employed for optimal procedural planning. Although the procedure was uneventful, risks such as device embolization, residual leak, or interference with valve function remain inherent to this technique. Careful patient selection, detailed preprocedural imaging, and precise device sizing are essential to minimize these complications.

## Supplementary Information

Supplementary file 1.

## Data Availability

No datasets were generated or analyzed during the current study.

## Abbreviations

AAP: Ascending aortic pseudoaneurysm
ASD: Atrial septal defect
CTA: Computed tomography angiography
SAVR: Surgical aortic valve replacement
