# Supplementary material for: Transcatheter closure of an ascending aortic pseudoaneurysm using an atrial septal defect occluder: a case report
Source: Egypt Heart J. 2025 Jun 16;77:62. doi: 10.1186/s43044-025-00663-x (PMC12170980; doi:10.1186/s43044-025-00663-x)
Supplement: Supplementary file 1 [file 43044_2025_663_MOESM1_ESM.pdf]

CARE case report guidelines

CARE Checklist of information to include when writing a case report

(cc) BY-NC-ND

Topic	Item	Checklist item description	Reported on Line
Title	1	The diagnosis or intervention of primary focus followed by the words "case report"	Title
Key Words	2	2 to 5 key words that identify diagnoses or interventions in this case report, including "case report"	Keywords
Abstract (no references)	3a	Introduction: What is unique about this case and what does it add to the scientific literature?	Abstract/Background
	3b	Main symptoms and/or important clinical findings	Abstract/Case presentation
	3c	The main diagnoses, therapeutic interventions, and outcomes	Abstract/Case presentation
	3d	Conclusion—What is the main "take-away" lesson(s) from this case?	Abstract/Conclusion
Introduction	4	One or two paragraphs summarizing why this case is unique (**may include** references)	Background, paragraphs 1-4
Patient Information	5a	De-identified patient specific information	N/A
	5b	Primary concerns and symptoms of the patient	Case presentation, paragraph 1
	5c	Medical, family, and psycho-social history including relevant genetic information	Case presentation, paragraph 1
	5d	Relevant past interventions with outcomes	Case presentation, paragraph 1
Clinical Findings	6	Describe significant physical examination (PE) and important clinical findings	Case presentation, paragraphs 1
Timeline	7	Historical and current information from this episode of care organized as a timeline	N /A
Diagnostic Assessment	8a	Diagnostic testing (such as PE, laboratory testing, imaging, surveys).	Case presentation, paragraphs 1,2
	8b	Diagnostic challenges (such as access to testing, financial, or cultural)	Case presentation, paragraphs 1,2
	8c	Diagnosis (including other diagnoses considered)	Case presentation, paragraphs 1,2
	8d	Prognosis (such as staging in oncology) where applicable	N /A
Therapeutic Intervention	9a	Types of therapeutic intervention (such as pharmacologic, surgical, preventive, self-care)	Case presentation, paragraph 3
	9b	Administration of therapeutic intervention (such as dosage, strength, duration)	Case presentation, paragraph 3
	9c	Changes in therapeutic intervention (with rationale)	N /A
Follow-up and Outcomes	10a	Clinician and patient-assessed outcomes (if available)	Case presentation, paragraph 4
	10b	Important follow-up diagnostic and other test results	Case presentation, paragraph 4
	10c	Intervention adherence and tolerability (How was this assessed?)	N /A
	10d	Adverse and unanticipated events	N /A
Discussion	11a	A scientific discussion of the strengths AND limitations associated with this case report	Background (No discussion part)
	11b	Discussion of the relevant medical literature **with references**	Background (No discussion part)
	11c	The scientific rationale for any conclusions (including assessment of possible causes)	Background (No discussion part)
	11d	The primary "take-away" lessons of this case report (without references) in a one paragraph conclusion	Conclusions
Patient Perspective	12	The patient should share their perspective in one to two paragraphs on the treatment(s) they received	N /A
Informed Consent	13	Did the patient give informed consent? Please provide if requested	Yes ☑ No ☐
